# Sitagliptin: Is It Effective in Routine Clinical Practice?

**DOI:** 10.1155/2015/950571

**Published:** 2015-05-18

**Authors:** Rita Mohan Dallumal, Siew Siang Chua, David Bin-Chia Wu, Shireene Ratna Vethakkan

**Affiliations:** ^1^Department of Pharmacy, Faculty of Medicine, University of Malaya, 50603 Kuala Lumpur, Malaysia; ^2^School of Pharmacy, Monash University Malaysia, Jalan Lagoon Selatan, 47500 Bandar Sunway, Selangor, Malaysia; ^3^Department of Medicine, Faculty of Medicine, University of Malaya, 50603 Kuala Lumpur, Malaysia

## Abstract

*Aim*. The present study was conducted to determine the glycaemic effects of sitagliptin in type 2 diabetes patients. *Methods*. Data was collected from patient medical records of a major teaching hospital in Malaysia, from 2009 to 2012. Glycated hemoglobin (HbA_1c_) values prior to and up to 12 months after the initiation of sitagliptin were analysed. The change in HbA_1c_ values was accounted for based on a generalized linear model generated using the Generalized Estimating Equations (GEE) method. *Results and Discussion*. Of the 457 patients, 53.6% were elderly and 81.4% were overweight. The mean HbA_1c_ (standard deviation) before initiation of sitagliptin was 8.5 (1.4)%. This dropped to 7.7 (1.4)%, 3 to 6 months after initiation of sitagliptin, with a mean difference of 0.8% (95% confidence interval (CI): 0.7–1.0; *P* < 0.001). However, this value increased to 8.0 (1.7)% after 7 to 12 months on sitagliptin (*P* = 0.002) with a mean difference from baseline of 0.6% (95% CI: 0.4–0.7; *P* < 0.001). *Conclusion*. In routine clinical practice, sitagliptin produces a significant reduction in mean HbA_1c_ (0.8%) within the first 6 months of use which corresponds to efficacy data obtained in controlled clinical trials. However, this reduction was lesser, 7 to 12 month later.

## 1. Introduction

The prevalence of diabetes is increasing worldwide. According to the World Health Organization (WHO), approximately 171 million people have diabetes globally, with 82 million in the Association of South East Asian Nations (ASEAN) region [1]. The International Diabetes Federation reported that 366 million people have diabetes in year 2011 and this figure is expected to increase to 552 million in 2030 [2]. According to the Malaysian National Health Morbidity Survey, the prevalence of diabetes in Malaysia has almost doubled among those aged 30 and above within a 10-year period, increasing from 8.3% in 1996 to 14.9% in 2006 [3]. Subsequently, the overall prevalence of diabetes in Malaysia has escalated to 22.9% [4].

The glycemic goal recommended by the Malaysian Clinical Practice Guidelines (CPG) is a glycated hemoglobin (HbA_1c_) of less than 6.5% (48 mmol/mol) but the American Diabetes Association (ADA) recommended a HbA_1c_  less than 7% (53 mmol/mol) [5, 6]. Most patients with type 2 diabetes do not achieve the HbA_1c_ target despite being on multiple medications. A study in Malaysia reported that only 17.4% (95% CI, 13.7 to 21.1%) of the patients achieved HbA_1c_ less than 6.5% (48 mmol/mol) [7]. Therefore, newer therapeutic agents such as dipeptidyl peptidase-4 (DPP-4) inhibitors have been introduced with the aim of achieving better glycemic control [8].

Sitagliptin (Januvia, Merck & Co. Inc., USA) is the first DPP-4 inhibitor marketed in the United States (USA) and was approved by the Food and Drug Administration of the United States (FDA, USA) in October 2006, for the treatment of type 2 diabetes [9]. The inhibition of DPP-4 leads to an increase in the active levels of incretins such as glucagon-like peptide-1 (GLP-1) and glucose-dependent insulinotropic polypeptide (GIP) which are involved in glucoregulation [10, 11]. Sitagliptin not only reduces the HbA_1c_ levels but also improves the fasting and postprandial plasma glucose [12–14].

Studies showed that sitagliptin reduced HbA_1c_ by 0.5 to 0.7% [8, 12, 13, 15]. However it has been suggested that DPP-4 inhibitors should be used only for patients with type 2 diabetes who are unable to tolerate other oral antidiabetes medications or who have not managed to achieve the glycemic target with the standard first-line agents [8, 16]. Sitagliptin confers several advantages in comparison to other antidiabetes medications as it is well tolerated, weight neutral and does not cause hypoglycemia [14, 17–19].

Most of the studies on the effectiveness of DPP-4 inhibitors are controlled trials [12, 14, 18]. However, data on the effects of this group of medications in clinical practice is still lacking although the demand for DDP-4 inhibitors has been increasing since its introduction into the market. Therefore, the present study was conducted to determine the glycemic effects of sitagliptin.

## 2. Materials and Methods

### 2.1. Patients and Setting

A retrospective study was conducted in a major teaching hospital in Kuala Lumpur, Malaysia. All patients prescribed with sitagliptin from 2009 to 2012 were identified from the Pharmacy Information System (PIS) of the hospital and data related to the patients were extracted from the patient medical records. The study was approved by the Medical Ethics Committee of the hospital (MEC reference number 890.32) prior to initiation of the study. A pilot study was conducted to assess the feasibility and practicability of the methodology as well as to ensure that the data collection form was able to gather all the information required to meet the study objectives.

Patients included were those with type 2 diabetes who were prescribed with sitagliptin by the teaching hospital within the study period. These patients must have obtained their sitagliptin supply from the hospital more than once and their medical records must be available for data extraction. Patients excluded were those who were on other DPP-4 inhibitors prior to the initiation of sitagliptin and patients prescribed with sitagliptin as first-line drug or were not started on sitagliptin by the teaching hospital under study since patients' data prior to the initiation of sitagliptin would not be available for comparison.

To analyze the effectiveness of sitagliptin, patients must have HbA_1c_ readings before and 3 to 6 as well as 7 to 12 months after the initiation of sitagliptin. Insulin must not be added to or discontinued from patients medications during the 12-month period.

### 2.2. Data Collection

A list of sitagliptin transactions in the teaching hospital was retrieved from its PIS. Patients who were dispensed with sitagliptin only once were excluded from the list. Duplicated registration numbers were then deleted, leaving only the first transaction of sitagliptin for each patient. The list of remaining patients' names with registration number was submitted to the Patient Information Department of the teaching hospital to retrieve the patient medical records. The medical records were screened and relevant information was extracted and recorded in a preprepared data collection form.

The primary outcome of this study was a change in HbA_1c_ values prior to and after the initiation of sitagliptin. Patients' HbA_1c_ values were collected at three points: at baseline prior to initiation of sitagliptin, 3 to 6 months, and subsequently 7 to 12 months after initiation of sitagliptin. Other information analyzed included demographic data of patients, medical condition(s), medication(s) and other clinical data before and after initiation of sitagliptin, reason(s) for initiating sitagliptin, and reported side effects or hypoglycaemia episodes.

### 2.3. Statistical Analysis

All data were entered into and analyzed using the IBM SPSS Statistics for Windows, version 20 (IBM Corporation, Armonk, NY). All data were subjected to descriptive analysis which generated frequencies and percentages. For numeric data, the mean (standard deviation) and median were also obtained.

Repeated measures of HbA_1c_ values obtained at three points during the one-year period from the same cohort of patients were assumed to be “dependent.” Therefore, a generalized linear model was generated to account for the change in HbA_1c_ values using the Generalized Estimating Equations (GEE) method. This model was used to assess any change in HbA_1c_ values with time (due to the initiation of sitagliptin) while controlling for patients' baseline characteristics. The equation is as follows:

(1) Yij=α+β1∗gender+β2∗age+β3∗race +β4∗marital  status+β5∗employment  status +β6∗number  of  years  of  diagnosis  with  diabetes +β7∗baseline  HbA1c +β8∗number  of  antidiabetes  agents  before  sitagliptin +β9∗change  in  regimen +β10∗initial  sitagliptin  dose +β11∗medication  prior  to  sitagliptin +β12∗sitagliptin  added  or  substituted+εij,

where *Y*_*ij*_ is the *i*th patient's HbA_1c_ value measured at the *j*th time point and *ε*_*ij*_ is the error term that cannot be explained by the model with *ε*_*ij*_ ~ *N*(0, *σ*2).

In addition, patients were divided into two groups: (1) patients with improvement in HbA_1c_ and (2) patients with no improvements in HbA_1c_ after using sitagliptin for 7 to 12 months. The same parameters as mentioned in the equation above were analysed using the GEE to test if there is any association between these parameters and the two groups of patients.

## 3. Results

### 3.1. Demographic and Baseline Characteristics of Patients

**Figure 1:**
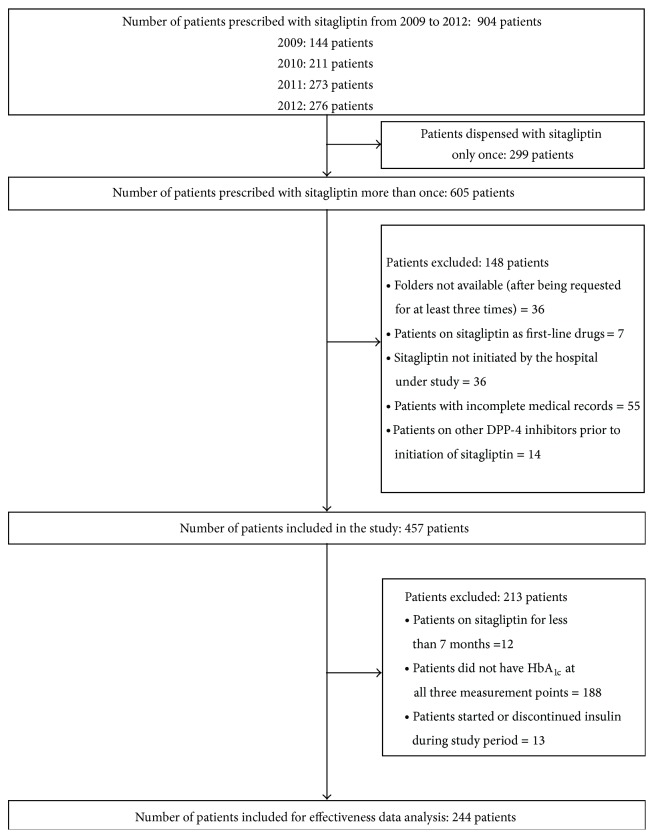
Inclusion of patients in the study.

A total of 904 patients were prescribed with sitagliptin at the major teaching hospital in Malaysia from 2009 to 2012. The number of patients dispensed with sitagliptin showed an increasing trend during the four-year period. However, only data from 457 patients who met the inclusion criteria was analyzed in this study (Figure 1).

Table 1 shows the demographic and clinical characteristics of the patients prior to the initiation of sitagliptin. At least half of the patients on sitagliptin (53.6%) were 65 years old and above. Three foreigners (a Burmese, an Eurasian, and an Iranian) were classified as “Others” under the ethnic groups. Amongst the patients who were still working, only six had health-related jobs.

At the time when the patients were started on sitagliptin, 93.8% had uncontrolled diabetes (defined as HbA_1c_ 6.5% (48 mmol/mol) and above) [5]. However, if based on the more commonly used HbA_1c_ target of 7% (53 mmol/mol) and above, 88.3% of the patients had uncontrolled diabetes. Most of the patients (78.4%) possessed a glucose meter at home and were able to monitor their own blood glucose level if desired.

Based on the body mass index (BMI), 81.4% of the patients were overweight (defined as BMI 23 kg/m^2^ and above) while 48.5% were obese (defined as BMI of 27.5 kg/m^2^ and above) [20]. If based on waist circumference, then 83% of the patients were classified as overweight (defined as ≥80 cm for female and ≥90 cm for male) [5].

Almost all the patients initiated on sitagliptin had diabetes for more than 5 years (94.8%). Most of the patients have other comorbidities (Table 1). Some patients also presented with complications of diabetes: 27 patients had nephropathy; 45 had retinopathy; 33 had neuropathy; 9 had diabetes foot ulcer; and 4 had amputation done.

Sitagliptin was prescribed to substitute patients' current antidiabetes medication in 19.2% of the cases whereas, in most cases (80.8%), sitagliptin was added to the current antidiabetes therapy. Sitagliptin was often added as an adjunct to metformin and sulphonylurea (42.3%).

The most common reason for the initiation of sitagliptin was uncontrolled blood glucose even though patients were already on other antidiabetes medications (82.6%). Intolerance to the side effects of other antidiabetes medications (6.1%) such as gastrointestinal disturbances (19 cases) which were associated with the use of metformin and acarbose was also reported. Other reasons were the occurrence of hypoglycemia (5.4%), concern on possible adverse effects of rosiglitazone on the cardiovascular system (2.6%), patients' unwillingness to start insulin (1.6%), nonadherence to insulin (4 patients), difficulty in obtaining rosiglitazone (2 patients), and request by a patient who claimed that sitagliptin has better effect on his blood glucose.

### 3.2. Effectiveness of Sitagliptin

**Figure 2:**
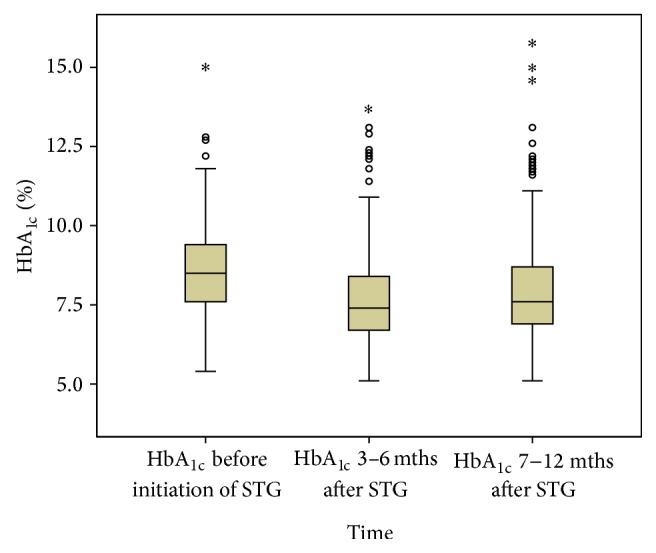
HbA_1c_  levels at all three measurement points (*N* = 244); o: outlier; ∗: extreme case; STG: sitagliptin; mths: months.

Effectiveness of sitagliptin was analyzed based on 244 patients whose HbA_1c_ levels could be obtained for all the three points (before initiation of sitagliptin, 3 to 6 months and 7 to 12 months after initiation of sitagliptin). Prior to the initiation of sitagliptin, the mean HbA_1c_ (standard deviation (SD)) was 8.5 (1.4)% (69 mmol/mol). After the initiation of sitagliptin, the mean HbA_1c_ value reduced significantly to 7.7 (1.4)% (61 mmol/mol) within the first 3 to 6 months (*P* < 0.001). Subsequently, after 7 to 12 months, the mean HbA_1c_ value was 8.0 (1.7)% (64 mmol/mol). This is significantly higher than that of the first 3 to 6 months (*P* = 0.002) but still significantly lower than before the initiation of sitagliptin (*P* < 0.001). The changes in HbA_1c_ before, 3 to 6 months and 7 to 12 months after initiation of sitagliptin are shown in Figure 2.

In the presence of missing data among the confounders, only 157 patients could be included in the generalized linear model. Due to the nature of the study design with repeated measures, there were 87 cases (35.7%) with incomplete covariates. These incomplete data were retained in the analysis using multiple imputation approach [21]. This means that all the 244 patients can be included using the imputed data as shown in Table 2. The model predicts that, on an average, a patient will experience a reduction in HbA_1c_ of 0.8% (95% confidence interval (CI): 0.7–1.0; *P* < 0.001) within the first 3 to 6 months after the initiation of sitagliptin and a reduction from baseline of 0.6% (95% CI: 0.4–0.7; *P* < 0.001) after being on sitagliptin for 7 to 12 months. This model also indicates that the patient's baseline HbA_1c_ value was the only factor which has a significant effect on the HbA_1c_ levels of patients after the initiation of sitagliptin.

Amongst the 244 patients included in determining the effectiveness of sitagliptin, 166 patients (68.0%) showed an overall reduction in HbA_1c_ after 7–12 months, whereas 69 patients (28.3%) showed an increase in HbA_1c_ and 9 patients (3.7%) had no change in HbA_1c_ after 7–12 months. The GEE analysis showed similar results when the patients were divided into two groups, that is, those with and without improvement in HbA_1c_ after being on sitagliptin for 7 to 12 months. Only patient's baseline HbA_1c_ value was associated with the two groups.

In addition, the proportion of patients who achieved the recommended glycemic target increased by twofold 7–12 months after the patient was on sitagliptin. After sitagliptin was initiated for 7–12 months, the number of patients who achieved HbA_1c_ below 6.5% increased from 16 patients (6.6%) at baseline to 29 patients (11.9%) (*χ* ^2^ = 41.886; *P* < 0.001) whereas the number of patients with HbA_1c_ below 7% increased from 28 patients (11.5%) at baseline to 68 patients (27.9%) (*χ* ^2^ = 25.160; *P* < 0.001).

### 3.3. Safety of Sitagliptin

The incidence of hypoglycemia reduced significantly from 61 patients (13.3%) to 40 patients (8.8%) (*χ* ^2^ = 7.591; *P* = 0.006) after the initiation of sitagliptin. Prior to the initiation of sitagliptin, the mean weight (SD) of 176 patients was 70.9 (17.2) kg. However, 12 months after the initiation of sitagliptin, the mean weight (SD) decreased to 70.4 (17.3) kg, that is, a mean decrease of 0.5 kg (95% CI: −0.02 to 0.96; *P* = 0.061) but was not statistically significant.

Sitagliptin was associated with side effects in eleven patients. Three patients had hypoglycaemia, whereas each of the remaining patients had worsening of allergic reaction, cough, weight loss, drowsiness, increase in creatinine level, swelling of the leg, and bloating and one patient just could not tolerate sitagliptin with no specific reason given.

## 4. Discussion

Sitagliptin is a DPP-4 inhibitor which is a relatively new group of antidiabetes medications in the market. Despite the lack of data on its effectiveness in clinical practice, its use has escalated since its introduction over the past few years. The number of patients prescribed with sitagliptin in the present study has doubled from 2009 to 2012.

Most of the patients on sitagliptin were 65 years old and above. This could be due to its potential benefits for causing minimal or no hypoglycemia in comparison to other antidiabetes medications such as the sulphonylureas [18, 19]. In addition, it is also more convenient due to its once daily oral dosing [18, 22].

A majority of the patients prescribed with sitagliptin were overweight based on their BMI or waist circumference as defined by the clinical practice guidelines [20]. Sitagliptin is preferred for overweight patients as it is weight neutral [12, 13, 17, 18]. This is an advantage over other antidiabetes medications such as the thiazolidinediones and sulphonylureas which are often associated with weight gain [18, 23]. However, this study did not demonstrate any significant change in body weight after the initiation of sitagliptin.

The mean (SD) duration of diabetes of patients prescribed with sitagliptin was 14.9 (7.6) years. This is similar to a study conducted in Taiwan although other studies had shown a shorter duration of 2 to 6 years [14, 17, 18]. Patients with a shorter duration of diabetes showed greater reduction in HbA_1c_ with the addition of sitagliptin; hence sitagliptin should be started earlier for better effect [18].

Sitagliptin was added to the existing antidiabetes regimens of most patients due to uncontrolled diabetes. This is consistent with that of other studies [9, 14, 24, 25]. This also accounts for the high baseline HbA_1c_ values in a majority of the patients (88.3% of the patients with HbA_1c_ > 7%) in the present study which was reported in another similar retrospective study [9]. Sitagliptin was usually prescribed for patients who were already on metformin and sulphonylurea. This means that sitagliptin was only initiated when the older groups of antidiabetes medications failed to produce adequate glycemic control. This is because sitagliptin is a relatively new antidiabetes agent and hence is reserved as an add-on therapy for patients who are unable to tolerate other antidiabetes medications or who have not reached the glycemic target with the standard first-line agents [8].

In the present study, 6.1% of the patients were started on sitagliptin when the other antidiabetes medications caused side effects. Sitagliptin is generally well tolerated with minimal adverse effects [14, 24]. This is a potential benefit of DPP-4 inhibitors as the occurrence of adverse effects often led to nonadherence to antidiabetes medications which in turn contributes to poor glycemic control [14]. It has been reported that patients on sitagliptin were less likely to discontinue their medications due to adverse reactions as compared to metformin monotherapy [26].

The Generalized Estimating Equations (GEE) model predicted that, on an average, sitagliptin resulted in a significant reduction of HbA_1c_ by 0.6% (95% CI: 0.4–0.7; *P* < 0.001) after 7 to 12 months. Studies have shown that sitagliptin reduced HbA_1c_ by 0.5% to 0.7% [8, 12, 13, 15]. However, the GEE model predicted a greater HbA_1c_ reduction 3 to 6 months after initiation of sitagliptin which is 0.8% (95% CI: 0.7–1.0; *P* < 0.001) compared to 7 to 12 months later. This indicates that sitagliptin produced the most glycemic effect during the first 6 months but this effect reduced significantly after that (*P* = 0.002) although still better than before the initiation of sitagliptin. This increment in HbA_1c_ may not be seen in studies which only had two-point measurements of HbA_1c_ (at baseline and at the end of the study). However, studies with more than two-point measurements showed similar effects with the use of sitagliptin [18, 27]. A study in Japan which reported similar outcomes attributed this increase to a reduction in compliance with diet and exercise therapy [28].

In addition, twice as many patients managed to attain glycemic control after the initiation of sitagliptin for 7 to 12 months. Other clinical studies showed similar results although they were carried out specifically to compare the efficacy of sitagliptin with placebo [12, 18]. One recent retrospective study which also assessed the effectiveness of sitagliptin in a clinical practice reported similar increase in the proportion of patients achieving glycemic control after using sitagliptin although the average reduction in HbA_1c_ is higher than that of the present study [29].

Mafauzy reported that 26.8% of patients with diabetes in public hospitals in Malaysia conducted self-monitoring of blood glucose [30]. On the contrary, 78.4% of the patients in the present study had a glucose meter at home. The difference may be attributed to the advance in technology from 2006 to 2012 and hence more patients have access to more convenient and cheaper glucose meters. The increase in the use of home glucose meters may also be due to an increase in awareness on the importance of self-monitoring of blood glucose.

There are several limitations in this study. Some data were not available as only information written in the patient medical records could be extracted. The dose of sitagliptin dispensed to the patients could not be standardized and this ranged from 25 to 100 mg, with most patients being on 100 mg. However, a study carried out in Japan showed that patients on sitagliptin 50 mg also showed a reduction in HbA_1c_ of 0.6% which is similar to that achieved by patients on sitagliptin 100 mg in other studies [31]. In the present study, a change in medications during and after the initiation of sitagliptin may occur but this was taken into account during the GEE model analysis which showed that changes in patients' medications did not significantly affect the change in HbA_1c_ levels. Patients' adherence to their medications could not be ascertained as no adherence assessment was carried out. Dietary habit and exercise could not be controlled, which may have affected the change in HbA_1c_.

## 5. Conclusion

In conclusion, the study provides evidence that sitagliptin produces a significant reduction of 0.8% in the mean HbA_1c_ value, 3 to 6 months after use. However, this reduction in HbA_1c_ was lesser 7 to 12 month later (0.6%) but still similar to that reported in clinical trials. Further investigations are required to determine if reduced adherence to sitagliptin is the reason for the increase in HbA_1c_ with prolonged usage of sitagliptin.

## Figures and Tables

**Table 1:** Baseline demographic and clinical characteristics of patients.

Patients characteristics	Number of patients (%)	Mean (SD) (median) [range]
Age (years) (*N* = 457)		
21–30	3 (0.7)	65 (11.7) (65) [23–100]
31–40	9 (2.0)	
41–50	33 (7.2)	
51–60	104 (22.7)	
61–70	152 (33.3)	
>70	156 (34.1)	
Gender (*N* = 457)		
Male	200 (43.8)	
Female	257 (56.2)	
Ethnic groups (*N* = 457)		
Malay	153 (33.5)	
Chinese	182 (39.8)	
Indian	119 (26.0)	
Others	3 (0.7)	
Marital status (*N* = 432)		
Single	21 (4.9)	
Married	392 (90.7)	
Divorced	2 (0.5)	
Widower	17 (3.9)	
Employment status (*N* = 329)		
Employed	115 (35.0)	
Unemployed	214 (65.0)	
Baseline HbA_1c_ (%) (mmol/mol)^a^ (*N* = 402)		
<6.5 (<48)	25 (6.2)	8.7 (1.7) (8.5) [4.9–17.1]
≥6.5 (≥48)	377 (93.8)	
<7.0 (<53)	47 (11.7)	
≥7.0 (≥53)	355 (88.3)	
Fasting blood glucose (mmol/L) (mg/dL)^b^ (*N* = 373)		
<4.4 (<79.2)	6 (1.6)	8.8 (3.2) (8.3) [2.9–36.9]
4.4–6.1 (79.2–109.8)	50 (13.4)	
>6.1 (>109.8)	317 (85.0)	
Presence of comorbidities^c^ (*N* = 457)		
Hypertension	379 (82.9)	
Dyslipidemia	322 (70.5)	
Cardiovascular disease	143 (31.3)	
Kidney disease	103 (22.5)	
Stroke	23 (5.0)	
Duration of diabetes (number of years) (*N* = 406)		
1–5	21 (5.2)	14.9 (7.6) (13) [0–41]
6–10	91 (22.2)	
11–15	118 (29.1)	
16–20	81 (19.9)	
>20	95 (23.4)	
Body weight (kg) (*N* = 294)		
<50	21 (7.1)	70.9 (19.1) (66.9) [36.8–195.0]
50–59	66 (22.5)	
60–69	84 (28.6)	
70–79	43 (14.6)	
≥80	80 (27.2)	
Body mass index (BMI) (kg/m^2^)^d^ (*N* = 140)		
<18.5	1 (0.7)	27.9 (5.4) (27.4) [17.2–45.4]
18.5–22.9	25 (17.9)	
23–27.4	46 (32.9)	
≥27.5	68 (48.5)	
Waist circumference (cm)^b^		
Male (*N* = 74)		95.5 (11.8) (96.0) [65.0–132.5]
<90	20 (27.0)	
≥90	54 (73.0)	
Female (*N* = 85)		93.8 (18.0) (91.0) [67–204]
<80	7 (8.2)	
≥80	78 (91.8)	
Number of diabetes medications (*N* = 449)		
1	94 (20.9)	2.12 (0.8) (2) [1–4]
2	224 (49.9)	
3	112 (25.0)	
4	19 (4.2)	
Type of antidiabetes medications (*N* = 449)		
Biguanide only	41 (9.1)	
Sulphonylurea only	43 (9.6)	
Insulin only	16 (3.6)	
Biguanide and sulphonylurea	186 (41.4)	
Biguanide and insulin	20 (4.5)	
Sulphonylurea and insulin	8 (1.8)	
Biguanide, sulphonylurea, and insulin	24 (5.3)	
Others^e^	111 (24.7)	
Initial dose of sitagliptin (*N* = 457)		
25 mg	34 (7.4)	
50 mg	158 (34.6)	
100 mg	265 (58.0)	

SD: standard deviation.

^a^HbA_1c_ is categorised based on the Clinical Practice Guidelines of Malaysia of <6.5% [5] and the American Diabetes Association of <7% [6].

^b^Fasting blood glucose and waist circumference are categorized based on the Clinical Practice Guidelines of Malaysia [5].

^c^Some patients may have more than one comorbidity.

^d^Body mass index (BMI) is categorized based on the Clinical Practice Guidelines on Management of Obesity [20].

^e^Others include combinations such as meglitinides, thiazolidinediones, and alpha-glucosidase inhibitors.

**Table 2:** Parameter estimates using the Generalized Estimating Equations (GEE) model (imputed) (*N* = 244).

Parameter	*B*	Std. error	95% Wald confidence interval		Hypothesis test		
			Lower	Upper	Wald chi-square	df	Sig.
(Intercept)	1.990	.5912	.831	3.149	11.326	1	.001
Years diagnosed							
>20 years	.467	.2467	−.016	.951	3.590	1	.058
16–20 years	.319	.2447	−.161	.798	1.697	1	.193
11–15 years	.366	.2301	−.085	.817	2.528	1	.112
6–10 years	.292	.2186	−.137	.720	1.780	1	.182
1–5 years	0^a^	—	—	—	—	—	—
Employment status							
No	−.118	.1402	−.393	.157	.707	1	.401
Yes	0^a^	—	—	—	—	—	—
Race							
Indian	−.116	.1250	−.361	.129	.865	1	.352
Chinese	−.201	.1122	−.421	.019	3.218	1	.073
Malay	0^a^	—	—	—	—	—	—
Marital status							
Widower	.163	.3932	−.607	.934	.173	1	.678
Divorced	.274	.2920	−.299	.846	.878	1	.349
Married	.136	.2674	−.388	.660	.259	1	.611
Single	0^a^	—	—	—	—	—	—
Gender							
Female	.047	.985	−.147	.240	.223	1	.637
Male	0^a^	—	—	—	—	—	—
Time of HbA_1c_ level							
7–12 months after sitagliptin	−.562	.0941	−.746	−.377	35.586	1	.000
3–6 months after sitagliptin	−.831	.0801	−.988	−.674	107.550	1	.000
Before initiation of sitagliptin (Baseline)	0^a^	—	—	—	—	—	—
Number of antidiabetes	−.038	.1301	−.292	.217	.084	1	.773
Age	−.010	.0065	−.023	.003	2.345	1	.126
Changes in regimen							
During initiation of sitagliptin	.101	.2169	−.324	.526	.216	1	.642
After initiation of sitagliptin	.020	.0981	−.172	.213	.043	1	.837
No change	0^a^	—	—	—	—	—	—
Initiation dose of sitagliptin							
100 mg	.057	.3022	−.536	.649	.035	1	.852
50 mg	.273	.2901	−.295	.842	.887	1	.346
25 mg	0^a^						
Medications prior to sitagliptin initiation							
Others	.281	.2851	−.278	.840	.969	1	.325
Biguanide, sulphonylurea, and insulin	.384	.3392	−.281	1.049	1.281	1	.258
Sulphonylurea and insulin	.009	.3411	−.660	.678	.001	1	.979
Biguanide and insulin	.226	.3094	−.381	.832	.533	1	.465
Biguanide and sulphonylurea	.194	.1855	−.170	.558	1.092	1	.296
Insulin alone	.140	.4808	−.802	1.083	.085	1	.770
Sulphonylurea alone	.062	.1920	−.314	.438	.104	1	.747
Biguanide alone	0^a^						
Initiation of sitagliptin							
Added to current regime	−.159	.1385	−.431	.112	1.318	1	.251
Switched from current regime	0^a^						
Baseline HbA_1c_	.790	.0407	.711	.870	377.159	1	000
(Scale)	1.114						

Dependent variable: HbA_1c_.

^a^Set to zero because this parameter is redundant.
